# Safety and Effectiveness of Aflibercept + Fluorouracil, Leucovorin, and Irinotecan (FOLFIRI) for the Treatment of Patients with Metastatic Colorectal Cancer (mCRC) in Current Clinical Practice: OZONE Study

**DOI:** 10.3390/cancers12030657

**Published:** 2020-03-11

**Authors:** Ian Chau, Marwan Fakih, Pilar García-Alfonso, Zdenĕk Linke, Ana Ruiz Casado, Eduardo Polo Marques, Pascaline Picard, Marina Celanovic, Thomas Cartwright

**Affiliations:** 1 The Royal Marsden NHS Foundation Trust, London and Surrey, Sutton SM2 5PT, UK; 2 City of Hope Comprehensive Cancer Center, Duarte, CA 91010, USA; mfakih@coh.org; 3 Hospital General Universitario Gregorio Marañón, 28003 Madrid, Spain; pgarcaalfonso@gmail.com; 4 University Hospital Motol, 150 06 Prague 5, Czech Republic; Zdenek.Linke@fnmotol.cz; 5 Hospital Universitario Puerta de Hierro-Majadahonda, 28222 Majadahonda, Spain; anaruizcasado@gmail.com; 6 Miguel Servet University Hospital, 50009 Zaragoza, Spain; eduardopolomarques@hotmail.com; 7 Ividata, 92300 Levallois Perret, France; Pascaline.Picard-ext@sanofi.com; 8 Sanofi, Cambridge, MA 02139, USA; marina.celanovic@sanofi.com; 9 Ocala Oncology, Ocala, FL 34474, USA; tcartwright4@cox.net

**Keywords:** aflibercept, antiangiogenic, clinical practice, FOLFIRI, metastatic colorectal cancer, observational

## Abstract

For patients with metastatic colorectal cancer (mCRC) that have failed a first-line oxaliplatin-based regimen, the preferred treatment option is an irinotecan-based regimen. This prospective, observational, noncomparative, post-authorization safety study (OZONE) evaluated the safety and effectiveness of aflibercept plus fluorouracil, leucovorin, and irinotecan (FOLFIRI) in patients with mCRC treated in daily practice after failure of an oxaliplatin-based regimen. Patients were grouped by age, renal impairment, hepatic impairment, race, number, and type of prior anticancer therapy. Of 766 treated patients enrolled, 59.5% were male, 94.8% had an Eastern Cooperative Oncology Group performance status of 0–1, all received previous chemotherapy (97.8% including oxaliplatin), and 58.6% had prior exposure to bevacizumab. At least one grade ≥ 3 treatment-emergent adverse event (TEAE) was reported in 68.3% of patients. Neutropenia, hypertension, diarrhea, and asthenia were the most frequently occurring grade ≥ 3 TEAEs. Antivascular endothelial growth factor class events were infrequent. Subgroup analyses did not reveal major differences in the safety profile according to age, renal and hepatic status, race, or prior anticancer therapy. For the total population, median overall survival was 12.5 months, median progression-free survival was 6.1 months, and overall response rate was 16.3%. Aflibercept in combination with FOLFIRI is a safe and efficacious regimen administered in current clinical practice to patients with mCRC previously treated with oxaliplatin. The study results, conducted in real-world clinical practice with a less selected patient population, are aligned with the VELOUR (NCT00561470) trial and no new safety issues were identified.

## 1. Introduction

Colorectal cancer (CRC) is the third most frequently diagnosed cancer and the second leading cause of cancer-related deaths worldwide [1]. Patients with metastatic disease (mCRC) have poor outcomes with a five-year survival rate of 13.8% [2]. First-line treatment recommendations include cytotoxic combinations of fluorouracil and leucovorin with irinotecan (FOLFIRI), oxaliplatin (FOLFOX), or oxaliplatin and irinotecan (FOLFOXIRI), or capecitabine plus oxaliplatin (XELOX). Chemotherapy regimens combined with antiepidermal growth factor receptor monoclonal antibodies, such as cetuximab and panitumumab, and anti-vascular endothelial growth factor (anti-VEGF) monoclonal antibodies, such as bevacizumab, are a standard of care in the first-line treatment of mCRC [3,4]. For patients with mCRC who progressed on an oxaliplatin-based regimen, the preferred treatment option is an irinotecan-based regimen, usually FOLFIRI. Combining antiangiogenic agents, which target the VEGF pathway, and cytotoxic chemotherapy is a recognized treatment strategy in this setting. Clinical efficacy of FOLFIRI plus anti-VEGF agents, such as aflibercept, bevacizumab, and ramucirumab, has been demonstrated in patients with mCRC [5,6,7,8]. 

VELOUR (NCT00561470) was a Phase III randomized, placebo-controlled study investigating aflibercept plus FOLFIRI for patients with mCRC who had disease progression on or after an oxaliplatin-based regimen. Aflibercept plus FOLFIRI significantly improved overall survival (OS; hazard ratio [HR] = 0.817; *p* = 0.0032), progression-free survival (PFS; HR = 0.758; *p* < 0.0001), and response rate (RR; *p* = 0.0001) versus placebo plus FOLFIRI. Based on these results, aflibercept, in combination with FOLFIRI, was approved for the treatment of patients with mCRC that are resistant to, or have progressed on, an oxaliplatin-containing regimen [9,10]. 

Whilst randomized controlled trials (RCTs) are crucial for evaluating the efficacy and safety of medical interventions [11,12], strict enrollment criteria often lead to the exclusion of elderly patients or those with comorbidities. Consequently, RCT populations may not fully represent cancer patients receiving treatment in routine clinical practice [12,13]. Observational studies evaluate the safety and effectiveness of therapies in a less selected population, ultimately providing information reflecting routine clinical practice [14].

The objective of this observational post-authorization safety study (PASS), OZONE, was to assess the safety and effectiveness of aflibercept plus FOLFIRI in patients with mCRC treated in daily practice after failure of an oxaliplatin-based regimen. Subpopulations defined by age, hepatic or renal impairment, race, and number and type of prior anticancer therapy were assessed. 

## 2. Methods

### 2.1. OZONE Study

OZONE was a prospective, multicenter, observational, noncomparative study evaluating patients with mCRC receiving aflibercept plus FOLFIRI (intravenous infusions) in the clinical setting after failure of an oxaliplatin-based regimen. Patients were followed for ≤ 24 months from aflibercept initiation or until death. OZONE was a PASS and thus registered in the European Union electronic Register of Post-Authorization Studies (EU PAS Register ENCEPP/SDPP/4836).

### 2.2. Site and Patient Selection

Physicians prescribing aflibercept plus FOLFIRI after failure of an oxaliplatin-based regimen were randomly selected based on physicians’ lists in each participating country. Independently in each country, sites offering participation in the study were randomly selected to ensure representativeness of the sample. The random process was stratified on country-specific criteria to accurately reflect routine clinical practices within each country. Patients enrolled in the study were prescribed aflibercept by their physician independently from study entry. Patients were excluded if they were participating in a clinical trial, receiving concomitant anti-VEGF agents and/or receiving aflibercept through an investigational clinical study or compassionate use program, or receiving aflibercept plus chemotherapy regimens other than FOLFIRI. This study was conducted in accordance with the principles outlined in the Declaration of Helsinki (18th World Medical Assembly, 1964) and all its subsequent amendments. Each patient provided signed, written, informed consent before enrolment.

### 2.3. Endpoints

There was no fixed study visit schedule, with visits occurring according to the treating physician’s clinical judgment. The primary objectives were to describe safety and clinical outcomes of aflibercept plus FOLFIRI in patients treated in daily practice for mCRC after failure of an oxaliplatin-based regimen. Data were analyzed based on the overall population and on patient subgroups as follows: age (< 65 and ≥ 65 years), renal function (impaired: creatinine clearance [CrCl] ≤ 80 mL/min and normal: CrCl > 80 mL/min), hepatic function (impaired: total bilirubin > upper normal limit [UNL], or aspartate transaminase [AST] or alanine aminotransferase [ALT] > 1.5 UNL and normal: total bilirubin ≤ UNL, or AST or ALT ≤ 1.5 UNL), race (Caucasian and non-Caucasian), number and type of prior anticancer therapy. Secondary objectives were to describe effectiveness (e.g., PFS, OS, RR) in subgroups and to describe utilization of health resources. PFS was defined as the time from the date of the first administration of aflibercept or FOLFIRI to the date of tumor progression or death due to any cause, whichever came first. OS was defined as the time interval from the date of first administration of aflibercept or FOLFIRI to the date of death due to any cause. Best overall response during treatment period was defined as complete response (CR), partial response (PR), stable disease (SD), progressive disease (PD), or not evaluable (NE). All evaluations were performed according to the participating institution’s practice and outcomes were based on the treating physician’s judgment. Results of laboratory tests that were systematically performed prior to chemotherapy were not collected. The study protocol did not include specific recommendations for standardized imaging evaluation, which was performed according to investigational sites protocols. 

Three categories of adverse events (AEs) were defined:

1. Pretreatment AEs: any AE reported during the pretreatment period.
2. Treatment-emergent AEs (TEAEs): an AE beginning, worsening, or becoming serious during the on-treatment period.
3. Post-treatment AEs: AEs reported during the post-treatment period.

Laboratory data collected at inclusion included a full blood count, biochemistry (including hepatic and renal function assessment), and urinary analyses. From the time period spanning from the informed consent signature to the first administration of aflibercept (pretreatment period), all AEs were collected and graded as per NCI-CTCAE version 4.03. During the interim period (post-initiation of aflibercept treatment), data was collected at approximately three-monthly (± 15 days) intervals throughout the observation period for a total period of 24 months or death, whichever came first. Dose adjustments were made in accordance with the relevant prescribing information. 

## 3. Results

### 3.1. Study Population

OZONE included 766 patients from 12 countries across Europe (84.3%) and North America (15.7%) (Table 1). Median age was 64 years, with 48.3% of patients aged ≥ 65 years and 13.6% aged ≥ 75 years. Most patients had an Eastern Cooperative Oncology Group performance status of 0–1 (94.8%), and renal and hepatic impairment was present in 35.0% and 19.6% of patients, respectively. Primary tumor site was the colon for 73.5% of patients and the rectum for 25.6%. All patients had metastatic disease at baseline, with 55.5% presenting with > 1 metastatic site. Mutated *KRAS* and *BRAF*, which are genes associated with poor prognosis in CRC, were present in 51.5% and 2.7% of patients, respectively. Over a third (35%) of patients received > 1 prior therapy for advanced disease. All patients received previous chemotherapy (97.8% including oxaliplatin) and 58.6% had prior exposure to bevacizumab. Median time from end of treatment to inclusion was 1.0 and 4.4 months in patients treated with advanced chemotherapy and neoadjuvant/adjuvant therapy, respectively. 

### 3.2. Treatment Exposure

The median duration of treatment was 16.4 (range 9.6–30.0) weeks (Table 2). Patients received a median of seven cycles (range 1–46) of treatment, including a median of six cycles with aflibercept. Relative dose intensities for aflibercept, irinotecan, and fluorouracil (5-FU) were 79.0%, 81.4%, and 82.9%, respectively. A total of 277 (36.2%) patients had ≥ 1 dose reduction or delay of aflibercept. 

### 3.3. Safety: Overall Population

At least one TEAE of any grade was reported in 98.3% of patients, with 68.3% of patients experiencing ≥ 1 grade ≥ 3 TEAE (Table 3 and Table 4). Neutropenia, hypertension, diarrhea, and asthenia were the most frequently occurring grade ≥ 3 TEAEs. Serious TEAEs were reported in 43.6% of patients, the most frequent of which were diarrhea, disease progression, and general physical health deterioration. TEAEs leading to death, excluding those related to disease progression, were reported in 14 (1.8%) patients, the most frequent of which were infections (*n* = 5), nervous system disorders (*n* = 3), and gastrointestinal (GI) disorders (*n* = 3). 

Anti-VEGF class events were infrequent, with GI perforation reported in 0.9% of patients (grouped term), GI fistula in 1.2%, fistula from other origin in 0.8%, and jaw osteonecrosis and posterior reversible encephalopathy syndrome in 0.4% each. Nephrotic syndrome was reported in one patient and no cases of thrombotic microangiopathy occurred.

### 3.4. Safety: Subpopulations

#### 3.4.1. Elderly

Among the overall population, 48.3% (*n* = 370) of patients were aged ≥ 65 years (Table 1). The rates of grade ≥ 3 TEAEs were similar between patients aged ≥ 65 and < 65 years (69.5% and 67.2%, respectively) (Table S1). Asthenia was more frequently reported (≥ 5%) in elderly versus younger patients. 

#### 3.4.2. Impaired Renal Function 

Among patients with renal status documented at baseline (*N* = 738), 34.9% (*n* = 258) had impaired renal function (CrCl ≤ 80 mL/min) (Table 1). Of patients defined as renally impaired, 16.7% (*n* = 43) and 83.3% (*n* = 215) had CrCl < 50 mL/min and 50–80 mL/min, respectively. Rates of all-grade and grade ≥ 3 TEAEs were similar between patients with and without renal impairment (Table S2). 

In patients with CrCl < 50 mL/min, grade ≥ 3 proteinuria was reported more frequently compared with the other two subgroups, whilst grade ≥ 3 asthenia was reported more frequently versus patients with CrCl > 80 mL/min. Hypertension was reported more frequently in the CrCl 50–80 mL/min and CrCl > 80 mL/min groups compared with the CrCl < 50 mL/min group (Table S2).

#### 3.4.3. Impaired Liver Function

Among patients with liver status documented at baseline (*N* = 657), 19.6% (*n* = 129) had impaired liver function (total bilirubin > UNL, or > 1.5 UNL) (Table 1). No grade ≥ 3 TEAEs were reported more frequently (≥ 5%) in either group (Table S3).

#### 3.4.4. Non-Caucasian 

Among patients with race documented at baseline (*N* = 762), 9.2% (*n* = 70) were non-Caucasian (Table 1). No grade ≥ 3 TEAEs were reported more frequently (≥ 5%) in either group (Table S4). 

#### 3.4.5. Received > 1 Prior Anticancer Therapy 

Among the overall population, 55.2% (*n* = 423) of patients had received > 1 prior anticancer therapy (Table 1). Grade ≥ 3 asthenia was reported more frequently in the > 1 prior line subgroup (Table S5). 

#### 3.4.6. Prior Exposure to Bevacizumab 

Among the overall population, 58.6% (*n* = 449) of patients had received prior treatment with bevacizumab (Table 1). Only grade ≥ 3 hypertension was more frequently reported in patients who had received no prior bevacizumab (Table S6). 

### 3.5. Efficacy

**Figure 1:**
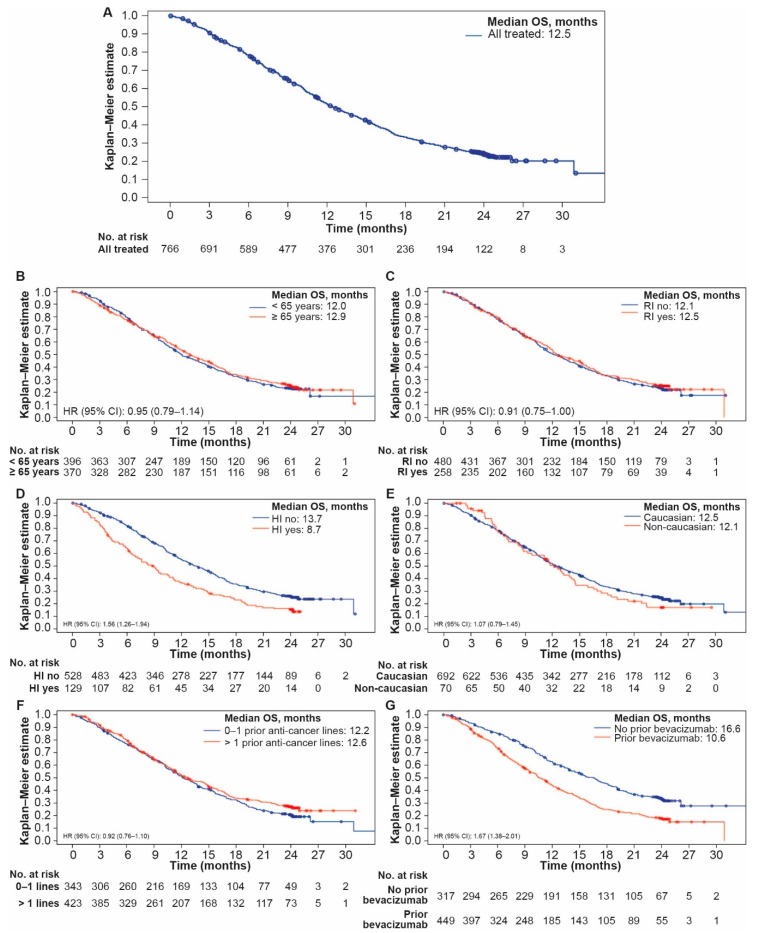
Overall survival (**A**) for the overall treated population; (**B**) according to age (< 65/≥ 65 years); (**C**) according to renal impairment (yes/no); (**D**) according to hepatic impairment (yes/no); (**E**) according to race (Caucasian/non-Caucasian); (**F**) according to prior anticancer therapy (0–1/> 1 lines); (**G**) according to prior use of bevacizumab (yes/no).CI, confidence interval; HI, hepatic impairment; HR, hazard ratio; OS, overall survival; RI, renal impairment.

**Figure 2:**
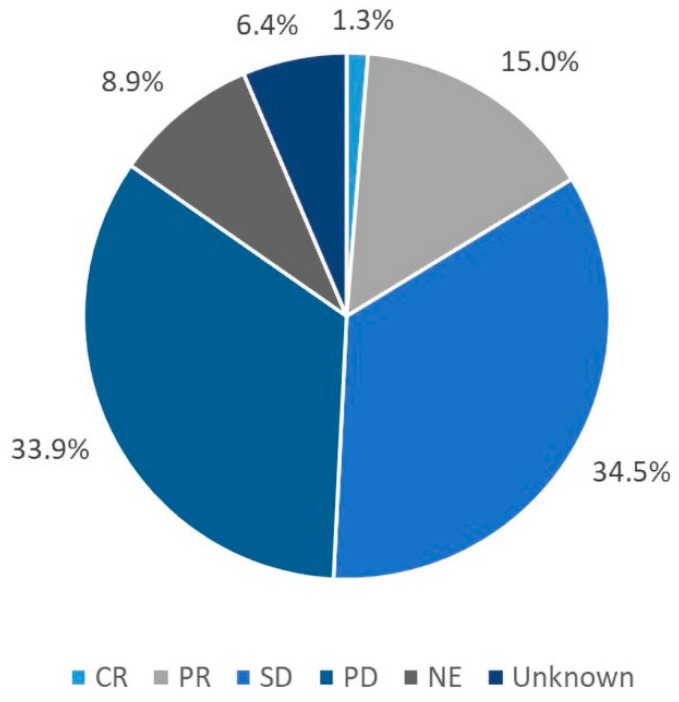
OZONE summary of overall response rate.CR, complete response; NE, not evaluable; PD, progressive disease; PR, partial response; SD, stable disease.

For the overall population, median OS was 12.5 months (Figure 1A), median PFS was 6.1 months (Figure S1), and overall RR (ORR) was 16.3% (Table 5). A CR was reported in 10 (1.3%) patients and 115 (15.0%) patients reported a PR (Figure 2). Multivariate analyses showed no clinically meaningful differences between groups defined by ages, race, baseline renal function, or number of prior lines of chemotherapy. Patients with hepatic impairment had shorter OS (8.7 vs. 13.7 months; HR [95% confidence interval {CI}]: 1.56 [1.26–1.94]), PFS (4.4 vs. 6.3 months; HR [95% CI]: 1.42 [1.16–1.75]), and ORR (10.9% vs. 17.0%; odds ratio [OR; 95% CI]: 0.63 [0.33–1.17]) versus those without hepatic impairment (Figure 1D, Figure S1D). Median OS in patients with prior bevacizumab therapy was 10.6 months versus 16.6 months in those with no prior bevacizumab treatment (HR [95% CI]: 1.67 [1.38–2.01]). Respective PFS and ORR were 5.2 versus 7. 5 months (HR [95% CI]: 1.59 [1.33–1.88]) and 11.5% versus 20.6% (OR [95% CI]: 0.51 [0.34–0.78]) (Figure 1G, Figure S1G). Patients aged < 65 years had lower ORR versus those ≥ 65 years (11.5% vs 19.2%; OR [95% CI]: 1.84 [1.21–2.81]) (Table 5). Similarly, patients without renal impairment had a numerically lower ORR versus those with renal impairment (13.5% vs. 19.4%; OR [95% CI]: 1.52 [0.99–2.33]). 

## 4. Discussions

In the VELOUR trial, aflibercept plus FOLFIRI was associated with prolonged OS and PFS versus placebo plus FOLFIRI in patients with mCRC [5]. Whilst studies such as these are required for registration purposes, Phase III RCTs have limitations, particularly when considering the rigorously controlled patient populations that are recruited, which often do not closely reflect patients in the clinic. PASSs evaluate the safety and effectiveness of therapies in a comparatively less selected population and are therefore useful for extending the knowledge of recently approved compounds in real-life practice, providing complementary information to formal clinical development [11,12,15,16,17,18,19]. OZONE evaluated the use of aflibercept plus FOLFIRI in routine clinical practice in patients with mCRC after failure of an oxaliplatin-based regimen, mirroring real-life patient management. The trial confirms that aflibercept in combination with FOLFIRI is a safe and efficacious regimen administered in current clinical practice to patients with mCRC previously treated with oxaliplatin. The study results, conducted in real-world clinical practice with a less selected patient population, are aligned with the VELOUR (NCT00561470) trial and no new safety issues were identified. 

Patients in the OZONE study were older than those in the VELOUR study (median 64 years, 48.3% aged ≥ 65 years versus median 61 years, 33.5% aged ≥ 65 years), although other disease characteristics did not differ. The proportion of patients who had neoadjuvant/adjuvant chemotherapy and who had prior exposure to bevacizumab was higher in OZONE versus VELOUR (45.7% vs. 26.5% and 58.6% vs. 27.6%, respectively), which is reflective of the difference in time between the initiation of both trials. Furthermore, patients in VELOUR had received only one prior line of therapy for metastatic disease. The OZONE population could have received > 1 line of therapy (55.2% had received > 1 prior line) and thus was consistent with the patients enrolled on more recent, real-world studies [15,16,17,18,19]. 

No new safety concerns emerged from this study. Diarrhea, asthenia, stomatitis, nausea, and hypertension were the most frequently occurring TEAEs of any grade, consistent with VELOUR and previously published real-world studies [5,15,16,17,18,19]. Anti-VEGF class effects were uncommon, and rates remained similar to those reported in VELOUR. 

Real-world studies also provide an opportunity to evaluate specific patient populations. Subgroup analysis of the OZONE study did not show major differences in the safety profile according to age, renal and hepatic status, race, or prior anticancer therapy. This supports the use of aflibercept in patients who are older and have renal or hepatic impairment, and independent of race or previous treatment with bevacizumab. However, these results should be interpreted with caution due to the small patient numbers investigated in several of the subgroups (*n* = 43 with CrCl < 50 mL/min; *n* = 70 non-Caucasian; *n* = 129 with hepatic impairment).

OZONE results support the conclusions of VELOUR and real-world studies, that aflibercept is a valuable treatment option for patients with mCRC [5,15,16,17,18,19]. When focusing on patient subgroups, OS multivariate analyses showed that treatment with aflibercept favored the subgroups of patients with no hepatic impairment and no prior use of bevacizumab. The observed numerical difference in median OS based on hepatic function may be impacted by the larger proportion of patients with baseline liver metastasis in the hepatic impairment subgroup (79.8% vs. 64.8% in patients with vs. without impaired hepatic function) and a greater proportion with metastatic disease at diagnosis (81.4% vs. 66.7%). Outcomes in patients who had prior bevacizumab (median OS: 10.6 months) are consistent with that reported in VELOUR (12.5 months) [20]. In real-world studies, the impact of prior bevacizumab is not as clear. In one study, prior bevacizumab was associated with numerically shorter OS and PFS [15]. However, the Spanish Named Patient Programme reported that prior bevacizumab was associated with improved PFS [17]. Restriction of antiepidermal growth factor receptor treatment to the wild-type KRAS population was implemented after the last patient completed treatment in the VELOUR study. This landmark change in the treatment of patients with mCRC may have contributed to the favorable 16.62-month median OS in the group of patients with no prior bevacizumab by selecting a wild-type KRAS population within this subgroup.

OZONE results further support the value of aflibercept for patients with mCRC in routine clinical practice and are consistent with what has previously been reported in a formal registration Phase III study [5]. However, there are some limitations, which are common to this type of study and must be considered. Although centers were selected at random, there are limitations regarding representativeness of the selected sites. No standardized method for evaluating efficacy could be used in the OZONE study, which affects the interpretation of RR and PFS. This should be considered when comparing with results from the VELOUR study, in which imaging evaluation was performed more frequently than in real-life practice. Results of the laboratory tests that were systematically performed prior to administration of chemotherapy were not collected in OZONE electronic case report forms, precluding comparison of hematologic toxicity with VELOUR. 

## 5. Conclusions

In conclusion, OZONE confirms that aflibercept in combination with FOLFIRI is a safe and efficacious regimen administered in current clinical practice to patients with mCRC previously treated with oxaliplatin. The study results, conducted in real-world clinical practice with a less selected patient population, are aligned with the VELOUR (NCT00561470) trial and no new safety issues were identified.

## Figures and Tables

**Table 1:** OZONE patient demographics.

Characteristic	Aflibercept/FOLFIRI (N = 766)
**Median age, years (range)**	64 (26–88)
Age ≥ 65, %	48.3
**Gender, %**	
Male	59.5
Female	40.5
**ECOG PS,^a^ %**	N = 746
0	49.6
1	45.2
2	4.8
3	0.4
**Race, %**	N = 762
Caucasian/white	90.8
Black	4.3
Asian/oriental	2.4
Other	2.5
**Country of origin, n**	
Czech Republic	80
France	165
Germany	22
Greece	34
Italy	69
Puerto Rico	4
Slovakia	6
Spain	210
Sweden	14
Switzerland	5
United Kingdom	41
United States	116
**Renal impairment,^b^ %**	N = 738
Yes	34.7
No	65.3
**Hepatic impairment,^c^ %**	*N* = 657
Yes	19.6
No	80.4
**Prior anticancer therapy, %**	
0–1 line	44.8
> 1 line	55.2
**Prior advanced disease prior anticancer therapy, %**	
0–1 line	65.0
> 1 line	35.0
**Prior neoadjuvant/adjuvant chemotherapy, %**	
Yes	45.7
No	54.3
**Prior bevacizumab, %**	
Yes	58.6
No	41.4
**Location of primary tumor, %**	
Colon	73.5
Rectum	25.6
Other	0.9
**Metastases site(s), %**	
Liver	67.5
Lung	51.4
Lymph nodes	22.5
Peritoneum	21.3
Other	21.0
***KRAS* status, %**	N = 765
Wild type	35.3
Mutated	51.5
Unknown	3.7
Not done	9.5
***BRAF* status, %**	
Wild type	22.5
Mutated	2.7
Unknown	16.7
Not done	58.1

ALT, alanine aminotransferase; AST, aspartate transaminase; CrCl, creatinine clearance; ECOG PS, Eastern Cooperative Oncology Group performance status; FOLFIRI, fluorouracil, leucovorin, and irinotecan; UNL, upper normal limit. ^a^ Scale used to assess patients’ level of functioning in terms of their ability to care for themselves, daily activity, and physical ability; ^b^ Defined as CrCl ≤ 80 mL/min; ^c^ Defined as either total bilirubin > UNL or AST or ALT > 1.5 UNL.

**Table 2:** OZONE study treatment exposure.

Treatment exposure parameter	Aflibercept/FOLFIRI (*N* = 766)
**Median duration of treatment, weeks (range)**	16.4 (2–108)
**Median number of treatment cycles, n (range)**	7 (1–46)
Aflibercept	6 (1–44)
Irinotecan	6 (0–46)
5-FU	6 (0–46)
**Median relative dose intensity, %**	
Aflibercept	79.0
Irinotecan	81.4
5-FU	82.9
**At least one dose modification, %**	
Aflibercept	36.2
Irinotecan	51.3
5-FU	67.0

5-FU, fluorouracil; FOLFIRI, fluorouracil, leucovorin, and irinotecan.

**Table 3:** OZONE overview of treatment-emergent adverse events.

TEAE, %	Aflibercept/FOLFIRI (*N* = 766)
Any TEAE	98.3
Any possible related TEAE	90.3
Any grade ≥ 3 TEAE	68.3
Any grade 5 TEAE	7.7
Any grade 3–4-related TEAE	50.1
Any serious TEAE	43.6
Any serious related TEAE	24.2
Any TEAE leading to death	7.8

FOLFIRI, fluorouracil, leucovorin, and irinotecan; TEAE, treatment-emergent adverse event.

**Table 4:** Summary of treatment-emergent adverse events: overall population.

TEAE	Aflibercept/FOLFIRI (*N* = 766)	
	All grade, %	Grade ≥ 3, %
**Overall TEAEs^a^**	98.3	68.3
Diarrhea	56.3	9.5
Asthenia	39.6	9.1
Stomatitis	37.9	5.2
Nausea	33.4	1.3
Hypertension	28.5	10.2
Neutropenia	24.7	15.1
Decreased appetite	22.7	2.7
Abdominal pain	21.7	3.8
Vomiting	21.5	1.8
Fatigue	21.0	3.5
Epistaxis	18.7	0.1
Constipation	16.6	0.1
Dysphonia	16.1	0.3
Alopecia	12.1	0.4
Proteinuria	11.7	2.7
Weight decreased	11.7	0.3
Anemia	10.7	1.3
Headache	10.4	0.3
**Overall serious TEAEs^b^**	43.6	36.8
Diarrhea	4.6	3.7
Disease progression	3.7	3.7
General physical health deterioration	3.3	3.1
Abdominal pain	2.9	2.2
Intestinal obstruction	2.5	2.2
Febrile neutropenia	2.2	2.2
Pyrexia	2.2	1.0

FOLFIRI, fluorouracil, leucovorin, and irinotecan; TEAE, treatment-emergent adverse event. ^a^ All-grade overall TEAEs reported in ≥ 10% of patients and associated grade ≥ 3 TEAEs; ^b^ All-grade serious TEAEs reported in ≥ 10% of patients and associated grade ≥ 3 serious TEAEs.

**Table 5:** Overall response rate multivariate analyses for subgroups of interest.

Response rate	Aflibercept/FOLFIRI (*N* = 766)
ORR, %	16.3
Age	
≥ 65 years	19.2
< 65 years	11.5
OR (95% CI)	1.84 (1.21–2.81)
Renal impairment	
Yes	19.4
No	13.5
OR (95% CI)	1.52 (0.99–2.33)
Hepatic impairment	
Yes	10.9
No	17.00
OR (95% CI)	0.63 (0.33–1.17)
Race	
Non-Caucasian	14.3
Caucasian	15.4
OR (95% CI)	0.31 (0.01–2.11)
Prior anticancer therapy	
> 1 line	14.4
0–1 line	16.2
OR (95% CI)	1.00 (0.66–1.52)
Prior bevacizumab therapy	
Yes	11.5
No	20.6
OR (95% CI)	0.51 (0.34–0.78)

CI, confidence interval; FOLFIRI, fluorouracil, leucovorin, and irinotecan; HR, hazard ratio; OR, odds ratio; ORR, overall response rate.

## Supplementary Materials

The following are available online at https://www.mdpi.com/2072-6694/12/3/657/s1, Figure S1: Progression-free survival A) for the overall treated population; B) according to age (< 65/≥ 65 years); C) according to renal impairment (yes/no); D) according to hepatic impairment (yes/no); E) according to race (Caucasian/non-Caucasian); F) according to prior anticancer therapy (0–1/> 1 lines); G) according to prior use of bevacizumab (yes/no), Table S1: Summary of treatment-emergent adverse events: by age, Table S2: Summary of treatment-emergent adverse events: by renal impairment, Table S3: Summary of treatment-emergent adverse events: by hepatic impairment, Table S4: Summary of treatment-emergent adverse events: by race, Table S5: Summary of treatment-emergent adverse events: by prior anticancer therapy, Table S6: Summary of treatment-emergent adverse events: by prior bevacizumab treatment.
