# Supplementary material for: Safety and Effectiveness of Aflibercept + Fluorouracil, Leucovorin, and Irinotecan (FOLFIRI) for the Treatment of Patients with Metastatic Colorectal Cancer (mCRC) in Current Clinical Practice: OZONE Study
Source: Cancers (Basel). 2020 Mar 11;12(3):657. doi: 10.3390/cancers12030657 (PMC7139359; doi:10.3390/cancers12030657)
Supplement: Supplementary file 1 [file cancers-12-00657-s001.pdf]

Supplementary figures and tables

Table S1. *Summary of treatment-emergent adverse events: by age*

TEAE	Aflibercept/FOLFIRI (N = 766)			
	Age < 65 years (n = 396)		Age ≥ 65 years (n = 370)	
	All grade, %	Grade ≥ 3, %	All grade, %	Grade ≥ 3, %
**Overall TEAEs[a]**	98.2	67.2	98.4	69.5
Diarrhea	54.5	8.1	58.1	11.1
Stomatitis	36.9	4.8	38.9	5.7
Asthenia	35.9	6.6	43.5	11.9
Nausea	34.6	1.3	32.2	1.4
Hypertension	30.3	11.4	26.5	8.9
Neutropenia	26.0	16.2	23.2	14.1
Vomiting	25.8	2.0	17.0	1.6
Abdominal pain	25.3	5.6	17.8	1.9
Fatigue	23.5	3.5	18.4	3.5
Constipation	21.5	0.3	11.4	0.0
Decreased appetite	20.2	2.5	25.4	3.0
Epistaxis	19.4	0.0	17.8	0.3
Dysphonia	16.2	0.3	15.9	0.3
Headache	13.4	0.5	7.3	0.0
Anemia	10.9	2.0	10.5	0.5
Weight decreased	10.9	0.5	12.7	0.0
Proteinuria	10.6	2.0	13.0	3.5
Alopecia	10.6	0.3	13.8	0.5
Pyrexia	10.4	1.3	8.4	0.8
Dyspnea	8.6	0.8	10.8	2.7

FOLFIRI, fluorouracil, leucovorin, and irinotecan; TEAE, treatment-emergent adverse event.

[a] All-grade overall TEAEs reported in ≥ 10% of patients in either subgroup and associated grade ≥ 3 TEAEs.

Table S2. Summary of treatment-emergent adverse events: by renal impairment

TEAE	Aflibercept/FOLFIRI (N = 738)					
	CrCl < 50 mL/min (n = 43)		CrCl 50–80 mL/min (n = 215)		CrCl > 80 mL/min (n = 480)	
	All grade, %	Grade ≥ 3, %	All grade, %	Grade ≥ 3, %	All grade, %	Grade ≥ 3, %
**Overall TEAEs[a]**	100.0	76.7	97.7	67.4	98.3	67.7
Diarrhea	51.2	11.6	60.0	8.4	54.8	9.6
Stomatitis	46.5	7.0	37.2	6.0	37.1	4.4
Asthenia	39.5	14.0	42.3	10.2	39.0	7.9
Decreased appetite	32.6	2.3	22.3	3.3	22.1	2.7
Neutropenia	23.3	14.0	21.4	13.0	26.3	16.0
Nausea	18.6	2.3	36.3	0.9	32.7	1.5
Anemia	16.3	0.0	10.7	0.9	9.6	1.5
Dysgeusia	16.3	0.0	3.7	0.0	4.6	0.0
Dysphonia	16.3	0.0	16.7	0.0	16.3	0.4
Vomiting	16.3	0.0	17.7	0.9	23.8	2.5
Pyrexia	14.0	2.3	7.4	0.9	10.2	1.0
Alopecia	14.0	0.0	12.6	1.4	11.7	0.0
Epistaxis	14.0	0.0	17.7	0.5	20.2	0.0
Proteinuria	11.6	7.0	13.5	2.8	11.5	2.5
Dyspnea	11.6	2.3	9.8	1.9	9.4	1.5
Hypokalemia	11.6	2.3	1.9	0.0	4.2	0.4
Constipation	11.6	0.0	12.6	0.0	18.3	0.2
Fatigue	11.6	0.0	19.5	4.2	21.5	3.8
Headache	11.6	0.0	7.9	0.5	11.9	0.2
Hypertension	11.6	0.0	28.4	11.2	30.4	11.0
Abdominal pain	9.3	2.3	20.9	1.9	22.7	4.8
Weight decreased	7.0	0.0	15.3	0.0	10.2	0.4

CrCl, creatinine clearance; FOLFIRI, fluorouracil, leucovorin, and irinotecan; TEAE, treatment-emergent adverse event.

[a] All-grade overall TEAEs reported in ≥ 10% of patients in any subgroup and associated grade ≥ 3 TEAEs.

Table S3. *Summary of treatment-emergent adverse events: by hepatic impairment*

	Aflibercept/FOLFIRI (N = 766)			
**TEAE**	**No hepatic impairment (n = 528)**		**Hepatic impairment (n = 129)**	
	**All grade, %**	**Grade ≥ 3, %**	**All grade, %**	**Grade ≥ 3, %**
**Overall TEAEs[a]**	98.1	66.3	98.4	75.2
Diarrhea	57.2	9.7	48.1	10.9
Asthenia	43.4	9.8	34.1	9.3
Stomatitis	38.6	4.9	33.3	3.1
Nausea	34.7	1.1	27.1	0.8
Hypertension	29.2	10.0	23.3	10.9
Decreased appetite	24.2	2.7	23.3	3.1
Neutropenia	23.7	13.6	25.6	15.5
Vomiting	22.2	2.3	23.3	1.6
Abdominal pain	21.4	4.2	24.0	4.7
Epistaxis	18.9	0.2	23.3	0.0
Dysphonia	18.4	0.4	11.6	0.0
Fatigue	17.8	3.6	21.7	3.9
Constipation	14.2	0.2	23.3	0.0
Proteinuria	13.1	2.7	7.0	1.6
Headache	12.3	0.2	4.7	0.8
Weight decreased	11.9	0.2	9.3	0.8
Anemia	11.7	1.3	8.5	1.6
Alopecia	11.4	0.4	15.5	0.8
Dyspnea	8.9	1.3	13.2	4.7
Back pain	7.8	1.1	10.1	0.8

FOLFIRI, fluorouracil, leucovorin, and irinotecan; TEAE, treatment-emergent adverse event.

[a] All-grade overall TEAEs reported in ≥ 10% of patients in either subgroup and associated grade ≥ 3 TEAEs.

Table S4. Summary of treatment-emergent adverse events: by race

TEAE	Aflibercept/FOLFIRI (N = 766)			
	Non-Caucasian (n = 70)		Caucasian (n = 692)	
	All grade, %	Grade ≥ 3, %	All grade, %	Grade ≥ 3, %
**Overall TEAEs[a]**	97.1	62.9	98.4	68.8
Diarrhea	54.3	7.1	56.2	9.5
Nausea	41.4	2.9	32.7	1.2
Stomatitis	34.3	8.6	38.3	4.9
Vomiting	31.4	2.9	20.7	1.7
Fatigue	31.4	5.7	20.1	3.3
Abdominal pain	27.1	5.7	21.2	3.6
Asthenia	25.7	7.1	40.6	9.2
Decreased appetite	17.1	1.4	23.4	2.9
Constipation	15.7	0.0	16.5	0.1
Hypertension	15.7	7.1	29.8	10.5
Palmar-Plantar	15.7	1.4	6.1	0.4
Dehydration	14.3	4.3	4.5	1.9
Epistaxis	14.3	0.0	19.1	0.1
Cough	14.3	0.0	5.8	0.0
Proteinuria	11.4	2.9	11.7	2.6
Peripheral edema	11.4	2.9	3.2	0.0
Pyrexia	11.4	0.0	9.2	1.2
Dizziness	11.4	0.0	4.2	0.1
Hypokalemia	10.0	2.9	3.3	0.3
Weight decreased	10.0	0.0	12.0	0.3
Headache	10.0	0.0	10.5	0.3
Back pain	10.0	0.0	7.5	1.2
Alopecia	8.6	1.4	12.6	0.3
Dysphonia	7.1	0.0	16.8	0.3
Dyspnea	5.7	2.9	10.1	1.6

FOLFIRI, fluorouracil, leucovorin, and irinotecan; TEAE, treatment-emergent adverse event.

[a] All-grade overall TEAEs reported in ≥ 10% of patients in either subgroup and associated grade ≥ 3 TEAEs.

Table S5. Summary of treatment-emergent adverse events: by prior anticancer therapy

	Aflibercept/FOLFIRI (N = 766)			
**TEAE**	**0–1 prior anticancer therapies (n = 343)**		**> 1 prior anticancer therapies (n = 423)**	
	**All grade, %**	**Grade ≥ 3, %**	**All grade, %**	**Grade ≥ 3, %**
**Overall TEAEs**[a]	98.3	66.5	98.3	69.7
Diarrhea	53.9	7.0	58.2	11.6
Asthenia	39.9	8.2	39.2	9.9
Stomatitis	39.4	5.8	36.6	4.7
Nausea	28.9	1.5	37.1	1.2
Hypertension	28.6	10.5	28.4	9.9
Neutropenia	26.8	16.0	22.9	14.4
Abdominal pain	22.2	5.0	21.3	2.8
Fatigue	20.4	3.2	21.5	3.8
Decreased appetite	20.4	2.9	24.6	2.6
Epistaxis	19.5	0.0	18.0	0.2
Vomiting	19.2	1.7	23.4	1.9
Dysphonia	16.9	0.6	15.4	0.0
Constipation	15.7	0.3	17.3	0.0
Proteinuria	14.3	2.6	9.7	2.8
Alopecia	12.0	0.0	12.3	0.7
Weight decreased	11.4	0.0	12.1	0.5
Pyrexia	11.1	1.7	8.0	0.5
Anemia	11.1	0.6	10.4	1.9
Headache	10.5	0.6	10.4	0.0

FOLFIRI, fluorouracil, leucovorin, and irinotecan; TEAE, treatment-emergent adverse event.

[a] All-grade overall TEAEs reported in ≥ 10% of patients in either subgroup and associated grade ≥ 3 TEAEs.

__Table S6.__ Summary of treatment-emergent adverse events: by prior bevacizumab treatment

	**Aflibercept/FOLFIRI (N = 766)**			
**TEAE**	**No prior bevacizumab (n = 317)**		**Prior bevacizumab (n = 449)**	
	**All grade, %**	**Grade ≥ 3, %**	**All grade, %**	**Grade ≥ 3, %**
**Overall TEAEs**[a]	99.4	70.0	97.6	67.0
Diarrhea	60.6	9.8	53.2	9.4
Asthenia	43.2	9.1	37.0	9.1
Stomatitis	39.4	4.4	36.7	5.8
Hypertension	36.3	14.8	22.9	6.9
Nausea	36.3	0.9	31.4	1.6
Neutropenia	27.1	17.0	22.9	13.8
Epistaxis	24.3	0.0	14.7	0.2
Vomiting	21.8	2.5	21.4	1.3
Abdominal pain	21.5	2.8	21.8	4.5
Decreased appetite	20.2	2.8	24.5	2.7
Dysphonia	20.2	0.3	13.1	0.2
Fatigue	17.0	1.3	23.8	5.1
Constipation	16.7	0.3	16.5	0.0
Proteinuria	13.9	2.5	10.2	2.9
Headache	13.6	0.3	8.2	0.2
Weight decreased	12.6	0.6	11.1	0.0
Anemia	12.3	0.3	9.6	2.0
Pyrexia	11.4	1.6	8.0	0.7
Alopecia	10.7	0.0	13.1	0.7
Dyspnea	8.5	1.6	10.5	1.6

FOLFIRI, fluorouracil, leucovorin, and irinotecan; TEAE, treatment-emergent adverse event.

[a] All-grade overall TEAEs reported in ≥ 10% of patients in either subgroup and associated grade ≥ 3 TEAEs.

Figure S1. *Progression-free survival* ***A)*** *for the overall treated population;* ***B)*** *according to age (< 65/≥ 65 years);* ***C)*** *according to renal impairment (yes/no);* ***D)*** *according to hepatic impairment (yes/no);* ***E)*** *according to race (Caucasian/non-Caucasian);* ***F)*** *according to prior anticancer therapy (0–1/>1 lines);* ***G)*** *according to prior use of bevacizumab (yes/no)*

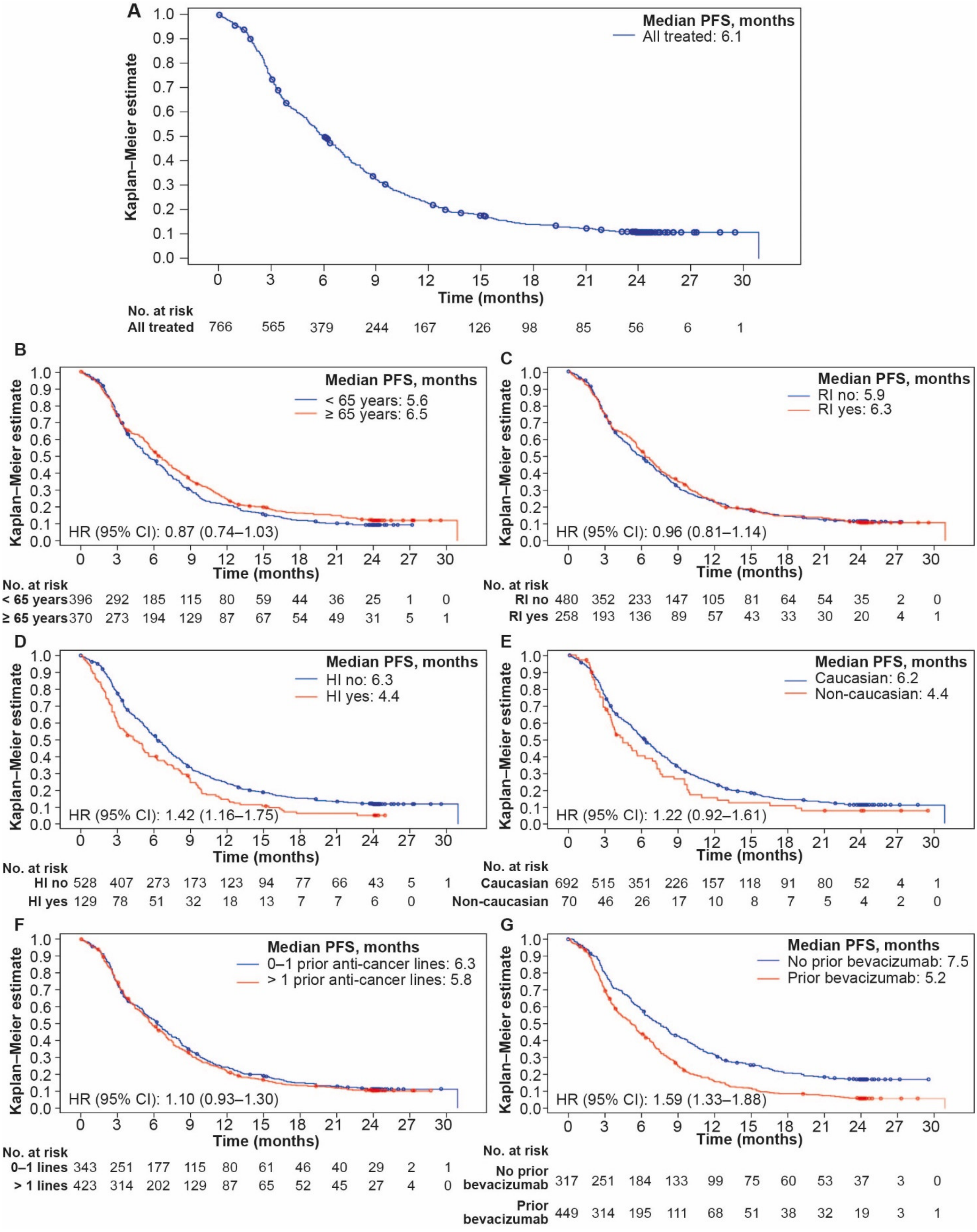

CI, confidence interval; HI, hepatic impairment; HR, hazard ratio; PFS, progression-free survival; RI, renal impairment.
